# Evaluating Teacher Language Within General and Special Education Classrooms Serving Elementary Students with Autism

**DOI:** 10.1007/s10803-021-05115-4

**Published:** 2021-06-09

**Authors:** Nicole Sparapani, Vanessa P. Reinhardt, Jessica L. Hooker, Lindee Morgan, Christopher Schatschneider, Amy M. Wetherby

**Affiliations:** 1 Present Address: School of Education and the MIND Institute, University of California, Davis, One Shields Ave., Davis, CA 95616 USA; 2 Present Address: Ontario, Canada; 3 Autism Institute, College of Medicine, Florida State University, 2312 Killearn Center Boulevard, Building A, Tallahassee, FL 32309-3524 USA; 4 Present Address: Marcus Autism Center, Children’s Healthcare of Atlanta, Department of Pediatrics, Emory University, 1920 Briarcliff Road, Atlanta, GA 30329-4010 USA; 5 Department of Psychology, Florida Center for Reading Research, Florida State University, 2010 Levy Avenue, Suite 100, Tallahassee, FL 32320 USA

**Keywords:** Autism spectrum disorder, Autism, Teacher language, Measurement invariance, Student characteristics

## Abstract

This study examined how teachers and paraprofessionals in 126 kindergarten-second grade general and special education classrooms talked with their 194 students with autism, and further, how individual student characteristics in language, autism symptoms, and social abilities influenced this talk. Using systematic observational methods and factor analysis, we identified a unidimensional model of teacher language for general and special education classrooms yet observed differences between the settings, with more language observed in special education classrooms—much of which included directives and close-ended questions. Students’ receptive vocabulary explained a significant amount of variance in teacher language beyond its shared covariance with social impairment and problem behavior in general education classrooms but was non-significant within special education classrooms. Research implications are discussed.

## Introduction

Teacher language is considered a unique and powerful resource for classroom learning, because interactive patterns directly impact student outcomes (Downer et al., 2010; Pianta, 2016). Studies have documented predictive associations between teacher language and student outcomes (e.g., Connor et al., 2020; Howes et al., 2008; Mashburn et al., 2008), with high quality interactions linked to academic growth (Curby et al., 2009; Hamre & Pianta, 2005), social competence (Mashburn et al., 2008; Wilson et al., 2007), and fewer problem behaviors (National Institute of Child Health and Human Development Early Child Care Research Network, 2003). Specific features of talk, including teachers’ use of open-ended questions, have also been associated with academic achievement and communication and language development (e.g., Burchinal et al., 2008; Milburn et al., 2014; Walsh, 2002). However, there has yet to be a thorough investigation of teacher language in elementary classrooms serving students with autism spectrum disorder (autism). With a growing prevalence (1 in 54; Maenner et al., 2020), complexity of learning needs (Fleury et al., 2014; Jones, 2015; Lindsay et al., 2013), and the push for inclusion (U.S. Department of Education, 2018), the need for understanding and evaluating effective educational practices for learners with autism is at a peak. We begin to address this need by examining the language environment, and the student characteristics that impact it, within general and special education classrooms serving students with autism. We posit that a thorough analysis of the language that students with autism experience in the classroom will provide a promising method for identifying and evaluating salient features of talk that can be woven into curricula to support student engagement and learning—an initiative that could offer insight into effective school-based practices and, at large, improve educational outcomes for learners with autism.

### Conceptualizing the Teacher Language Construct

Examining the language that teachers use with their students with autism is complicated by differences in the operationalization of teacher language as a construct across educational settings and populations. Hence, we draw from the early childhood, general education, and special education literature to guide our conceptualization of teacher language in relation to students with autism. To begin, many studies evaluating teacher language have been carried out in early childhood general education classrooms, with investigations conceptualizing and measuring teacher language as an aspect of broader interaction quality as outlined on the *Classroom Assessment Scoring System* (CLASS; Pianta et al., 2008). The CLASS is a widely-used observation scale for quantifying teacher-student interactions in the classroom by measuring the quality of teachers’ feedback, content, and questions to students. High quality teacher-student interactions, in which teachers provide explicit and genuine feedback to students, model academically rich vocabulary, and ask open-ended questions to encourage critical thinking have been associated with positive student outcomes such as academic achievement and social emotional development (Burchinal et al., 2008; Hamre & Pianta, 2005, 2007). This literature base, although broad, is rigorous as it utilizes large samples, quantitative and consistent measures of interaction quality, and a range of student outcome measures (O’Connor, 2010; Pianta et al., 2008).

Other studies examining teacher language have also used observational methods to capture the amount and type of language that teachers direct to their students. This method for measuring teacher language quantifies specific features of talk, such as the frequency of open-ended questions, close-ended questions, language models, and directives. However, conceptualization of and tools for measuring teacher language in this manner have varied across studies and environments. In a few studies within early childhood settings, teacher language has been categorized by pragmatic function, such as language used to encourage continued interaction (open- and close-ended questions), to provide information (language models), and to direct behavior (directives; DeWitt & Hohenstein, 2010; Gast et al., 2010; Walsh & Rose, 2013). Studies that have categorized teacher language by pragmatic function have been descriptive in nature, documenting differences in the talk that teachers use across educational contexts. For example, Gast et al. (2010) found that teachers most frequently provided information during unstructured activities, such as snack and free-play, and encouraged interaction and directed behavior during academically-based activities.

Across settings, studies have also conceptualized teacher language as use of open-ended language to encourage interaction and close-ended language to elicit specific information (Walsh, 2002; Westgate & Hughes, 1997). These studies have primarily described the language environment, examining the relation between features of teachers’ talk and students’ communication (Connor et al., 2020; Jadallah et al., 2011; Liu, 2008; Sadler & Mogford-Bevan, 1997; Sparapani et al., 2020). Studies have found that teachers’ use of open-ended language, such as asking open-ended questions and making contextual statements is associated with student participation (e.g., Milburn et al., 2014), generative or interactive talk (Connor et al., 2020; Duke et al., 2011; Reznitskaya et al., 2009), and on-topic initiations (Mercer, 1996; Walsh, 2002). Open-ended language has also been linked with higher order thinking and academic achievement (Burchinal et al., 2008; Connor et al., 2020).

In contrast, teachers’ use of close-ended language, such as asking yes/no, choice, and simple “wh” questions, using expectant pauses and fill-in strategies, and directing student behavior have been associated with less generative talk and fewer instances of on-topic initiations (Milburn et al., 2014; Sadler & Mogford-Bevan, 1997). While close-ended language may help to scaffold interactions (Mirenda & Donnellan, 1986), this type of talk often elicits fixed or constrained responses, potentially limiting opportunities for students to contribute new ideas and think critically about a given topic (Milburn et al., 2014; Turnbull et al., 2013; Walsh, 2002). Similarly, studies have ssuggested that teachers’ use of directives may limit student engagement within activities and the overall quality of the interaction (de Kruif et al., 2000; Liu, 2008; McWilliam et al., 2002; Williford et al, 2017).

Sparapani et al. (2020) used systematic observational methods to quantify the amount and type of language that interventionists used with elementary students with autism during a reading intervention. Although this study was conducted outside the classroom, the authors operationalized four categories of teacher language drawn from the research literature: responsive language, open-ended questions, close-ended questions, and directives. The authors found that interventionists’ use of open-ended questions was associated with students’ generative responding, while close-ended questions and directives were associated with less frequent generative responding and initiating. These findings are consistent with the literature outlined above linking features of teachers’ talk to students’ contributions, providing some evidence that current conceptualizations of the teacher language construct may extend to students with autism. However, understanding the dimensionality of teacher language for students with autism, and how and whether specific features of talk should be measured separately or together, would provide a more consistent method for conceptualizing and assessing the construct in future studies.

### The Influence of Student Characteristics on Teacher Language

This study is informed by dynamic systems and bioecological theories (Bronfenbrenner & Morris, 2006; Sameroff, 2009; Yoshikawa & Hsueh, 2001), which highlight learning as a dynamic and transactional process between students and their environment. This framework posits that learning is a dynamic and transactional process involving multiple sources of influence that work together to shape child development over time, with a core emphasis on the bidirectional interplay between the child and his/her environment (Connor, 2016; Sameroff, 2009). We can apply this framework to illustrate the dynamic and transactional interplay between students and their environment by studying how teachers talk with their students, and how individual students (with a range of cognitive, language, and social abilities) influence the amount and types of talk that teachers use. That is, while studies suggest that teacher language influences and shapes students’ participation and development (e.g., Burchinal et al., 2008; Mashburn et al., 2008), we posit that the individual characteristics that students bring with them into the classroom influence the language teachers use with them, which in theory, shapes their classroom learning experiences and development over time.

There is an emerging literature base outlining the effect of specific student characteristics on teacher language within classrooms serving students with and without autism. Studies have documented differences in the frequency of open- versus close-ended questions based on children’s age, with teachers using fewer open-ended questions with younger typically developing children (Girolametto & Weitzman, 2002). Teachers’ perceptions of problematic behavior have also been found to influence the type of talk they use with their students, with studies suggesting that teachers use higher rates of directives to comply with students they perceive as “misbehaving” (Dobbs & Arnold, 2009; Dobbs et al., 2004; Koenen et al., 2019; Partee et al., 2020). Similarly, studies examining students with autism within preschool and clinical settings have suggested that autism symptom severity, receptive and expressive language ability, cognitive functioning, and perceived problematic behavior affect the amount and/or type of language teachers use in educational settings (e.g., Dykstra et al., 2013). More specifically, studies have shown that teachers use less language overall with students who exhibit more severe autism symptoms and/or cognitive and language impairment (Dykstra et al., 2013; Irvin et al., 2013, 2015). Teachers also tend to be less responsive with students who exhibit challenging behaviors and those with co-occurring cognitive and language impairment (Keen et al., 2005; Qian, 2018). For example, Irvin et al. (2015) examined the association between adult talk and student characteristics during center activities in 73 children with autism participating in inclusive preschool settings and found that adults used more language to manage behavior with students who exhibited more severe autism symptoms. Similarly, Sparapani et al. (2020) examined interactions between elementary students with autism and their interventionists during a reading intervention and found that interventionists used more language to direct behavior (e.g., sit down, don’t touch that, etc.) than all other types of language with their students who exhibited more severe autism symptoms and limited expressive language skills. The interventionists within the study also asked fewer questions overall, none of which were open-ended, to students with limited expressive language abilities.

This emerging body of literature highlights the importance of teacher language on student learning experiences and the need for continued research, particularly with studies carried out in elementary classrooms serving students with autism. Taken together, these studies suggest immense variability in the language that students with autism experience in the classroom, and these differences may be, in part, due to individual student characteristics (Bronfenbrenner & Morris, 2006; Connor, 2016; Dykstra et al., 2013). It is possible that the types of language that teachers use, such as open-ended questions and language models, may afford different learning opportunities for different students (Hestenes et al., 2004; Irvin et al., 2013; Sparapani et al., 2020). However, better understanding the learning opportunities that varying types of teacher language afford is an area of future research.

### Study Purpose and Research Aims

Previous studies examining teacher language have largely conceptualized the construct by measuring key aspects of language associated with the quality of teacher-student interactions or by categorizing teacher language into varying dimensions, such as pragmatic function or open- verse close-ended statements. Structural equation modeling (SEM) has been utilized in larger studies examining teacher-student interaction quality (e.g., Hamre et al., 2013, 2014); however, to our knowledge, no study has utilized SEM to examine the latent structure of teacher language in classrooms serving students with autism or whether differences in the language environment exist between general and special education settings. Thus, this study utilized SEM (multi-group confirmatory factor analysis) to characterize dimensions of teacher language as outlined in the literature, evaluate similarities and differences in the dimensions of teacher language between general and special education settings, and examine the predictive association between student characteristics and teacher language within a large sample of students with autism and their educators.

## Methods

### Participants

This study included participants who were recruited between 2010 and 2014 for the Classroom Social Communication Emotion Regulation Transactional Support (SCERTS) Intervention project (CSI; Morgan et al., 2018), a randomized controlled intervention trial evaluating the efficacy of the SCERTS Model as a teacher-mediated intervention for learners with autism. Teachers and students were recruited from 54 schools across eight school districts in FL, GA, and San Diego, CA. Data for the present study include classroom video observations recorded at the beginning of the school year, prior to the start of intervention during Years 1–3 of the CSI project. See Morgan et al., 2018 for a complete description of participant recruitment. This study was approved by the Institutional Review Board at Florida State University as well as the review boards for participating school districts.

Teachers and their paraprofessionals from 126 kindergarten–second grade general (*n* = 71) and special education (*n* = 55) classrooms participated in the study. While lead teachers are primarily responsible for students’ attainment of curricular standards, language provided by all members of each student’s educational team was analyzed for this study. This approach was considered to be more ecologically valid as all adults in the classroom may interact with students throughout the school day (e.g., Irwin et al., 2015). Therefore, the focus of this study was on the language environment that each student experienced rather than individual teachers’ language.

Students who participated in the larger trial met the following criteria: (1) enrollment in kindergarten, first, or second grade at the beginning of the school year in either general or special education classrooms; (2) confirmation of a clinical or educational diagnosis of Autistic Disorder, PDD-NOS, or Asperger Syndrome as defined by the DSM-IV-TR (American Psychiatric Association [APA], 2000) prior to the start of the study; and (3) no co-occurring severe motor delay/impairment, dual sensory impairment, or history of traumatic brain injury.

Participants in this study (*N* = 194) included 78 students whose primary placement was in general education classrooms and 116 students with primary placements in special education classrooms. However, during the baseline video collection, nine students were in an educational setting that differed from their primary classroom placement (i.e., student primarily receives services within a special education setting but spends a percentage of time in a general education classroom). This resulted in a total of 87 students participating in general education classrooms and 107 students participating in special education classrooms during baseline. Twenty-nine percent of the students (*n* = 57) were in kindergarten, 41% (*n* = 80) in first grade, and 29% (*n* = 57) in second grade. The sample was primarily male, consistent with the observed 4:1 sex ratio for individuals with autism (Baio et al., 2018). See Table 1 for student demographic information.

**Table 1:** Student demographics by classroom setting

	Total (n = 194)	General education (n = 78)	Special education (n = 116)
Demographics			
Age, M (SD)	6.76 (1.00)	6.80 (0.91)	6.73 (1.05)
Gender (male)	85.10%	84.60%	85.30%
Race			
White	64.40%	69.20%	61.20%
Black	12.90%	11.50%	13.80%
Asian	7.70%	2.60%	11.20%
Multiracial	5.70%	3.80%	6.90%
NR	9.30%	12.80%	6.90%
Ethnicity			
Hispanic	22.20%	17.90%	25.00%
NR	9.30%	12.80%	6.90%
Grade			
Kindergarten	29.40%	26.90%	31.00%
First	41.20%	43.60%	39.70%
Second	29.40%	29.50%	29.30%

### Measures

As part of the larger CSI project (Morgan et al., 2018), students completed a battery of diagnostic and developmental measures at the beginning of the school year that examined: (1) autism symptomology; (2) intellectual functioning; (3) adaptive functioning; (4) receptive and expressive vocabulary; (5) social impairment; and (6) internalizing and externalizing behaviors. Descriptive statistics on each of the standardized measures for students in general and special education classrooms are presented in Table 2. Each month, for the duration of the study, trained videographers also collected a continuous 60-min video observation of each student participant in their classroom. See Morgan et al., 2018 for a complete description of the video observation process. This study specifically examined video observations collected during baseline, prior to the start of intervention as part of the larger CSI project.

**Table 2:** Student developmental characteristics by classroom setting

	General education		Special education		
	M	SD	M	SD	*p* value
ADOS-2 CSS (*n*s = 78, 116)					
Social Affect	6.97	1.84	6.97	1.95	0.999
RRB	7.09	2.36	7.72	1.79	0.049
Total	7.08	1.87	7.31	1.84	0.393
SB-5 (*n*s = 78, 116)					
Nonverbal Scales	8.45	3.74	5.58	4.43	> .001
Verbal Scales	6.21	3.54	3.14	2.56	> .001
Abbreviated IQ	83.92	18.55	66.51	18.3	> .001
VABS-II (*n*s = 68, 106)					
Communication	83.53	13.47	72.3	12.44	> .001
Socialization	77.94	10.31	68.69	9.30	> .001
Daily Living	83.85	14.45	74.14	12.28	> .001
ABC	79.82	11.24	70.44	9.69	> .001
PPVT-4 (*n*s = 72, 111)	85.69	16.86	66.44	24.21	> .001
EOWPVT-4^a^ (*n*s = 76, 111)	106.32	11.56	93.00	13.50	> .001
SRS (ns = 76, 105)	67.25	9.01	67.11	10.38	0.927
ASEBA TRF (*n*s = 76, 108)					
Internalizing	60.38	9.92	57.52	10.69	0.064
Externalizing	59.32	8.43	60.34	7.63	0.062
Total	63.24	8.14	63.19	7.04	0.979

Student classroom setting based on primary classroom placement

*ABC* adaptive behavior composite, *ASEBA* Achenbach System of Empirically Based Assessments, *ADOS* Autism Diagnostic Observation Schedule, *CSS* calibrated severity score, *EOWPVT-4* Expressive One-Word Picture Vocabulary Test, *PPVT-4* Peabody Picture Vocabulary Test, *RRB* Restricted and Repetitive Behaviors, *SB-5* Stanford Binet, *SRS* Social Responsiveness Scale, *TRF* Teacher Report Form, *VABS-II* Vineland Adaptive Behavior Scales

^a^Standard scores computed from larger sample’s z scores

#### Autism Diagnostic Observation Schedule

The Autism Diagnostic Observation Schedule (ADOS-2; Lord et al., 2002) is a semi-structured behavior observation comprising several different activity modules measuring autism symptoms. Because of variability with regard to age and language level, students were administered Module 1, 2, or 3. To allow for comparison of scores across modules, calibrated severity scores based on previous validation studies (Gotham et al., 2009; Hus et al., 2014) were estimated for the Social Affect and Restricted and Repetitive Behavior subscales and the Total score. The ADOS is considered the “gold standard” measure for determining autism diagnostic status.

#### Stanford-Binet Intelligence Scale

The Stanford-Binet Intelligence Scale, Fifth Edition (SB-5; Roid, 2003) is a standardized measure evaluating intellectual ability in a broad age range of individuals. For this study, the verbal and nonverbal routing subtests of the SB-5 were administered to derive an abbreviated IQ (ABIQ). The SB-5 demonstrates good internal consistency and reliability (coefficient values 0.95–0.98) based on its validation on a large nationally representative sample of children.

#### Vineland Adaptive Behavior Scales

The Vineland Adaptive Behavior Scales, Second Edition (VABS-II; Sparrow et al., 2005) is a structured caregiver interview that assesses adaptive functioning in elementary-age students across three domains: Communication, Daily Living Skills, and Socialization. In addition, the VABS-II includes an Adaptive Behavior Composite (ABC), which provides an overall estimate of an individual’s adaptive behavior. The VABS-II has been found to have strong reliability and validity, with split half reliability estimates ranging from 0.91 to 0.97 based on the normative subsample of children ages 5–9.

#### Peabody Picture Vocabulary Test

The Peabody Picture Vocabulary Test, Fourth Edition (PPVT-4; Dunn & Dunn, 2007) is a norm-referenced measure for assessing receptive vocabulary in a broad age range of individuals and yields standard scores. Reported reliability coefficients for the PPVT-4 range from 0.89 to 0.95 based on its validation on a large nationally representative sample of children.

#### Expressive One Word Picture Vocabulary Test

The Expressive One Word Picture Vocabulary Test, Fourth Edition (EOWPVT-4; Brownell, 2000) is a standardized measure for assessing expressive vocabulary skills in individuals from early childhood through adulthood and yields standardized score. The EOWPVT-4 was normed on a large nationally representative sample, showing strong internal consistency (αs = 0.93–0.98) and test–retest reliability (corrected *r*s = 0.88–0.97). At the time of baseline evaluation, a group of students in this study sample attained raw scores (*n* = 29) below the possible standard score range of the EOWPVT-4. This was addressed as part of the larger study by computing standard scores from the z-scores for the larger study sample (see Morgan et al., 2018). Age was included in analyses as a covariate, as the EOWPVT was normed by age.

#### Social Responsiveness Scale

Teachers completed the Social Responsiveness Scale (SRS; Constantino & Gruber, 2005), which characterizes the presence and severity of social skill impairment associated with autism. Total T-scores are based on a mean of 50 and standard deviation of 10, with higher scores indicating greater social impairment. Reported coefficient alphas for the teacher-report form range from 0.96 for female children to 0.97 for male children, demonstrating strong internal consistency.

#### Teacher Report Form

The Teacher Report Form (TRF), a component of the Achenbach System of Empirically Based Assessment (Achenbach & Rescorla, 2001), is a teacher-report measure of emotional problems, maladaptive behavior, and academic behavior in students aged 6–18 years. The TRF compromises two comprehensive composites represented as T-scores (*M* = 50, *SD* = 10): Internalizing Behaviors and Externalizing Behaviors. The TRF demonstrates strong reliability, with reported reliability coefficients greater than 0.90. It is important to note 24% (*n* = 47) of the students were under 6 years of age, with 53% (*n* = 25) of this subgroup at or above 5.50 years old, however, teachers completed the age 6–18 form for all students to allow for continuity of the measure across the sample.

#### Observational Methods—Categories of Teacher Language

Trained coders used Noldus Observer® Video-Pro Software to identify a 15-min sample comprised of three different 5-min activities (e.g., 5:00 mathematics + 5:00 literacy + 5:00 transition) from the 60-min video observation following the procedures described in Sparapani et al. (2016). They next identified each instance that teachers directed the following six observable categories of language to an individual student or group of students: open-ended questions (questions that do not have predetermined answers), language models (contextual statements and expansions), close-ended questions (questions that elicit a specific response), directives (directing students’ behavior to comply), indirect requests (questions that imply a behavioral response), and fill-ins (pausing to elicit responses). See the Appendix for the definitions and coding specifications of the teacher language categories. Interrater agreement between coders was first established using percent agreement with a minimum criterion of 80% agreement across 10 consecutive video observations. Interrater agreement was then calculated on 20% of the data using percent agreement and Cohen’s kappa for each of the teacher language categories, yielding an average percentage score of 83% and kappa coefficient score of 0.75.

### Analytical Methods

#### Model Specification and Identification

Specification of three models of teacher language was guided by the literature. We first evaluated teacher language as a unidimensional construct, with all six observed indicators loading onto one factor. Although this model is not outlined in the literature, it represents the most parsimonious structure of teacher language. We next evaluated a two-factor model, which included teachers’ use of open-ended language to encourage interaction (open-ended questions and language models) and close-ended language to elicit specific information (close-ended questions, directives, indirect requests, and fill-ins). Finally, we evaluated a three-factor model conceptualized as language used to encourage continued interaction (open-ended questions, close-ended questions, and fill-ins), provide information (language models), or direct behavior (directives and indirect requests). Confirmatory factor analysis (CFA) was used to evaluate the absolute and relative fit of the three models using Mplus software (Muthén & Muthén, 1998) with the ‘complex’ feature to account for the nested nature of the data (Kline, 2016). Each model met the recommended identification assumptions. The model degrees of freedom (*df*) were greater than zero and scaling constraints were imposed on the variances of the latent factors and loadings of the error terms. The three-factor model was identified by fixing the error term of the single indicator factor to equal 1- *r* (S^2^) (Kline, 2016).

#### Multi-group Confirmatory Factor Analysis

Differences in the structure of teacher language across general and special education classrooms were evaluated using a multi-group CFA. We first examined each of the three teacher language models independently for both general and special education settings in order to identify a common model with adequate to good fit for each classroom setting. We used weighted least squares-mean and variance adjusted (WLSMV) estimation due to the smaller sample size and inclusion of continuous and non-continuous variables in the models (Kline, 2016). Evaluation of model fit was guided by information gleaned from the following fit statistics: root mean square error of approximation (RMSEA) and comparative fit index (CFI), with values greater than 0.95 indicating good fit; and the χ^2^/*df* index, with values less than two indicating good fit (Hoyle, 2012; Kline, 2016). Data analysis was conducted at the student level.

Once a common model with adequate to good fit was identified across both classroom settings, a baseline model without cross group factor constraints was fit, followed by a model with full cross group constraints on the factor loadings and covariances. Using WLSMV estimation, we compared the fit of the baseline model to a model with full-cross group constraints. Differences between nested models were examined with the DIFFTEST function in Mplus, which rescales the χ^2^ values estimated with WLSMV (Muthén & Muthén, 1998). A significant difference indicates full or partial measurement invariance and the need to examine specific group differences in factor loadings and covariances. Partial measurement invariance models were examined by freeing constrained parameters individually, comparing each model with the baseline model until there was a nonsignificant difference between the partially constrained model and the baseline model. In the final step, we tested for latent factor variance equality between the general and special education classrooms by comparing the partial invariance measurement model to a model with cross group constraints on the latent factors.

#### Structural Equation Modeling

Structural equation modeling was conducted to examine the unique contribution of students’ receptive vocabulary, severity of social impairment, and presence of problem behavior to the teacher language latent factor across general and special education classrooms (separately). Students’ receptive vocabulary measured with the PPVT, severity of social impairment measured with the SRS, and presence of internalizing and externalizing behaviors measured with the TRF were added to the model as predictors of teacher language. It is important to note that we included students’ receptive vocabulary as a means to gauge their understanding of oral language. We did not include measures of intellectual functioning (*r* = 0.81) and expressive vocabulary (*r* = 0.86) because they were highly correlated with receptive vocabulary, and this multi-collinearity would have jeopardized model fit and increased the number of estimated parameters overall. It is important to note that statistically significant path coefficients (β) within the model indicate unique variance in explaining teacher language over and beyond shared or common variance among the three predictors (Nagy et al., 2006). A nonsignificant path coefficient might be associated with teacher language; however, the covariance shared with the other predictors reduces its unique contribution to the teacher language factor. Analyses were conducted using Mplus software and the WLSMV estimator, and the complex feature was used to account of the nested structure of the data.

## Results

### Sample Descriptive Statistics

The sample of students showed marked variability in intellectual functioning (*M* = 73.51, *SD* = 20.25) and overall adaptive behavior (*M* = 74.10, SD = 11.25), with 59% (*n* = 114) of the students exhibiting ABIQ scores at or above 70. Expressive vocabulary (based on sample z-scores), on average (*M* = 98.41, *SD* = 14.31), was higher than receptive language (*M* = 74.02, *SD* = 23.54), which is consistent with research examining language functioning in individuals with autism (Charman et al., 2003; Howlin et al., 2004). We observed significant differences between classroom setting for developmental characteristics but not for autism symptom severity (ADOS-2), severity of social impairment (SRS), and presence of problem behaviors (TRF).

### Teacher Language Within General and Special Education Classrooms

Teachers in special education classrooms used more verbal bids overall (*M* = 66.55; *SD* = 37.10) compared to teachers in general education classrooms (*M* = 45.63; *SD* = 26.64), with a higher proportion of their bids directed toward individual students (*M* = 76.26%; *SD* = 23.53) compared to groups of students (*M* = 23.53%; *SD* = 24.10). Teachers in general education classrooms demonstrated the opposite pattern, with 59.16% (*SD* = 30.57) of their bids directed toward groups of students. Special education teachers also used a higher proportion of language models (*M* = 13.07%; *SD* = 10.55) compared to general education teachers (*M* = 6.47%; *SD* = 6.09). Across both classroom settings, teachers used relatively more directives and close-ended questions than all other types of talk and very few open-ended questions, fill-ins, and indirect requests. See Table 3.

**Table 3:** Teacher language descriptive information by classroom setting

	General education				Special education				
	Instances^a^		Percentage^b^		Instances^a^		Percentage^b^		
	*M*	SD	*M*	*SD*	*M*	*SD*	*M*	*SD*	
Open-ended questions	2.49	(3.00)	6.27	(9.48)	1.21	(1.91)	2.08	(3.95)	
Language models	6.47	(6.09)	16.58	(12.52)	13.07	(10.55)	22.59	(13.95)	
Close-ended questions	15.60	(10.90)	33.76	(15.01)	20.85	(15.20)	30.60	(14.54)	
Directives	14.28	(11.29)	32.77	(17.87)	24.36	(15.69)	38.38	(17.56)	
Indirect requests	2.91	(4.08)	5.72	(5.94)	2.91	(3.77)	4.34	(4.69)	
Fill-ins	2.76	(3.42)	5.76	(6.27)	2.77	(3.73)	3.90	(4.90)	
Total verbal bids	45.63	(26.64)	–	–	66.55	(37.10)	–	–	
Individual bids	20.07	(22.79)	40.78	(30.47)	50.83	(33.68)	76.26	(23.53)	
Group bids	25.52	(18.80)	59.16	(30.57)	15.58	(20.52)	23.53	(24.10)	

^a^Number of observed instances

^b^Percentage of Total Verbal Bids by classroom setting

Pearson product-moment correlations among the types of teacher talk and standardized measures were estimated across classroom settings (Table 4). Overall, we observed small to moderate positive, significant correlations between the teacher language categories in both general and special education classrooms. Within general education classrooms, teachers’ use of open-ended questions was positively associated with students’ expressive vocabulary (*r* = .23), and directives and indirect requests were positively associated with autism symptom severity (*r* = .22; *r* = .24). Teachers’ use of close-ended questions and fill-ins was negatively associated with students’ cognitive functioning (*r* = − .31), receptive vocabulary (*r* = − .28), and expressive vocabulary (*r* = − .35). However, these relations looked different in special education classrooms, such that, teachers were more likely to ask open-ended questions with their students who exhibited less severe autism symptoms (*r* = − .26), and they used more directive language with students who exhibited less developed expressive vocabulary (*r* = − .36), receptive vocabulary (*r* = − .31), and cognitive abilities (*r* = − .36). Similar to general education classrooms, teachers used more directive language with their students with more severe autism symptoms (*r* = .21).

**Table 4:** Pearson correlations among the teacher language categories and standardized measures across classroom setting

	1	2	3	4	5	6	7	8	9	10	11	12	13
1. Open-ended	–	.26**	.19*	− .08	.14	.09	.21*	.01	.06	.18	.15	− .26**	− .08
2. Language models	.13	–	.36***	.21*	.36***	.24*	.39***	.33***	− .02	− .08	− .12	− .03	− .03
3. Close-ended	.23*	.37***	–	.35***	.39***	.30**	.41***	.53***	.12	.18	.16	− .05	.01
4. Directives	.02	.28**	.30**	–	.24**	.15	− .01	.78***	− .36***	− .31**	− .36***	.21**	.04
5. Indirect requests	.23*	.19	.34***	.35***	–	.35***	.26**	.30**	.05	.08	.04	.04	.11
6. Fill-ins	.35***	.25*	.35***	.02	− .01	–	.48***	.15	− .01	.10	− .01	.05	.15
7. Group bids	.34***	.35***	.35***	.28**	.14	.50***	–	− .23**	.22*	.24*	.22*	− .24*	− .10
8. Individual bids	.05	.37***	.55***	.49***	.52***	.10	− .18	–	− .26**	− .21*	− .26**	− .26**	.08
9. ABIQ	.13	− .09	− .31**	.15	− .10	− .22*	.03	− .27**	–	.81***	.84***	− .51***	− .26**
10. PPVT	.19	− .17	− .28**	− .03	− .16	− .22*	.04	− .37**	.69***	–	.85***	− .44***	− .14
11. EOWPVT	.23*	− .19	− .35***	− .07	− .20	− .22*	− .02	− .38**	.69***	.86***	–	− .42***	− .16
12. SRS	− .12	.09	.05	.22*	.24*	− .07	.01	.23*	− .10	− .13	− .14	–	.61***
13. TRF	.11	.01	.15	.20	.18	− .06	.01	.17	.03	.17	.09	.68***	–

The values above the diagonal represent the observed correlations for the special education classrooms. The values below the diagonal represent the observed correlations for the general education classrooms

*p < .05; **p < .01; ***p < .001

### Multi-group Confirmatory Factor Analyses

#### Data Preparation

Across both classroom settings, we observed low frequencies of open-ended questions, fill-ins, and indirect requests during the classroom observation, resulting in a high proportion of zeros for these categories (open-ended questions = 47%; fill-ins = 38%; indirect requests = 26%). See Table 3. These variables were dichotomized for analysis to address the substantial disproportion in the frequencies of the behaviors and prevent model fitting errors. The total loss of information as a result of dichotomize was assumed to be minimal (McCallum et al., 2002). Language models, close-ended questions, and directives were normally distributed (skewness and kurtosis ± 2.00).

#### Model Results

The one-, two-, and three-factor models evidenced excellent fit to the data for special education classrooms; however, for general education classrooms, the two-factor model did not converge and the three-factor model exhibited extreme collinearity between two of the three factors (encourage continued interaction and direct behavior; *r* = 0.906). Thus, the one-factor model evidenced the best fit to the data for both general education (RMSEA = 0.067 [0.001–0.147]; CFI = 0.920; χ^2^/*df* = 1.386) and special education classrooms (RMSEA = 0.015 [0.095–0.110]; CFI = 0.996; χ^2^/*df* = 1.023). For both classroom settings, each of the factor loadings were significantly different from zero (*p* < 0.05), excluding open-ended questions (*p* = 0.160) within general education classrooms*.*

#### Measurement Invariance

**Fig. 1:**
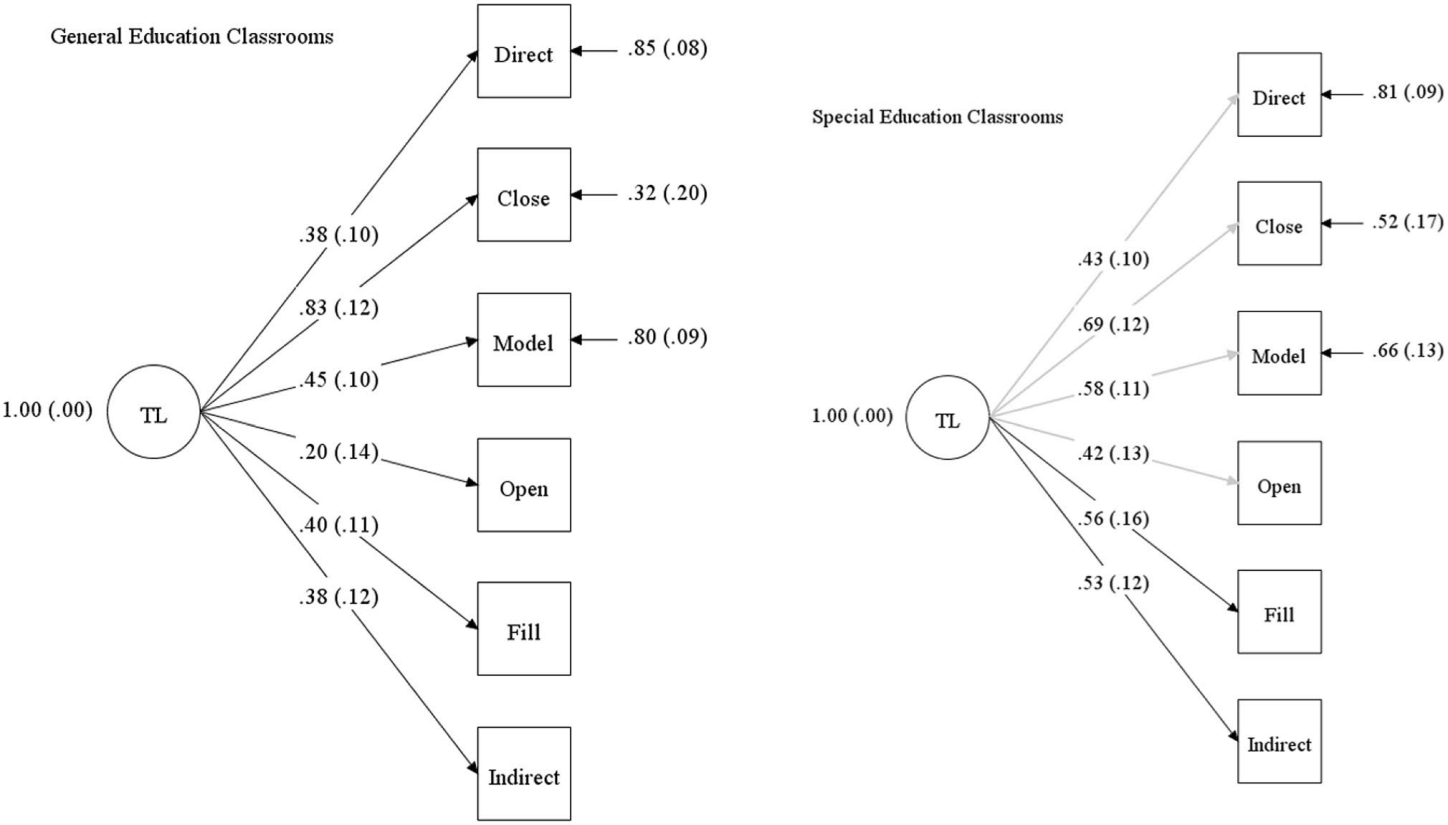
Partial measurement invariance testing of teacher language between general and special education classrooms. Teacher language (TL); directives (Direct); close-ended questions (Close); language models (Model); open-ended questions (Open); fill-ins (Fill); indirect requests (Indirect). Partial measurement invariance between the classroom settings was established by freeing the following factor loadings (grey): directives, close-ended questions, models, and open-ended questions

After identifying the one-factor model as the common model for both classroom settings, we created a model without cross group constraints (baseline model), which showed overall adequate fit to the data (RMSEA = 0.066 [0.095–0.117]; CFI = 0.917; χ^2^/*df* = 1.4180. We then compared the fit between the baseline model with a model containing full cross group constraints (χ^2^ = 111.798 [*df* = 27]) using the DIFFTEST function. Findings indicated a significant difference between the two models (Δ χ^2^ = 67.461 [*df* = 7], *p* < 0.001), suggesting differences in the patterns of the factor loadings between general and special education classrooms. We next established partial measurement invariance by individually freeing parameters and comparing each model with the baseline model (without cross group constraints) until there was not a significant difference between the partially constrained and baseline models. This resulted in freeing the following factor loadings: directives, close-ended questions, models, and open-ended questions (Δ χ^2^ = 6.01 [*df* = 2], *p* = 0.05). When comparing this partial invariance model to a model with cross group constraints on the latent factors, we observed a significant difference in the latent factors between the general and special education classrooms (*p* > 0.05). Overall, results indicate partial metric invariance of the one-factor model in that there was an equivalent underlying structure of teacher language across classroom settings, however, the degree to which the language categories were explained by this underlying language factor differed by classroom setting. See Fig. 1.

#### Structural Equation Modeling

Given the significant differences we observed in student developmental characteristics and the structure of teacher language between classroom settings, we analyzed the general and special education SEM models separately in order to understand how students’ characteristics influenced teachers’ language across setting. The SEM model evidenced good fit for the data in general education classrooms (RMSEA = 0.033 [0.000–0.097]; CFI = 0.955; χ^2^/*df* = 1.088) and adequate fit for special education classrooms (RMSEA = 0.055 [0.000–0.104]; CFI = 0.883; χ^2^/*df* = 1.309). Within general education classrooms, students’ receptive vocabulary explained a significant amount of variance in teacher language (β = –0.31; *p* < 0.01) over and above its shared covariance with social impairment and problem behavior. The path coefficient for teacher-reported problem behavior approached statistical significance (β = 0.27; *p* = 0.11); social impairment was non-significant and β = –0.12; *p* = 0.48). SEM results of the special education classroom group indicated non-significant path coefficients for receptive vocabulary (β = 0.14; *p* = 0.31), social impairment (β = 0.16; *p* = 0.18), and problem behavior (β = 0.01; *p* = 0.98). Although not statistically significant, the associations between receptive vocabulary and social impairment with teacher language in special education classrooms were positive, yet they were negative in the general education classrooms.

## Discussion

Teacher language has been proposed as an important intervention target for supporting classroom learning and student development (Pianta, 2016), yet this phenomenon in relation to students with autism is poorly understood. This study utilized classroom video observations to examine the amount and type of language that teachers used with their students with autism as well as SEM to understand the factor structure of the teacher language construct within general and special education classrooms. Findings from this study support our theoretical framework that incorporates dynamic systems and bioecological theories, making three primary contributions to the literature. It offers a detailed, descriptive analysis of the language environment that kindergarten–second grade students with autism experience in classrooms while highlighting differences in the measurement model of teacher language between general and special education settings. These data also provide empirical evidence for conceptualizing the language that students with autism receive within general and special education classrooms, which may afford a consistent method for evaluating and monitoring teacher language in relation to student outcomes in future research. Finally, these findings extend the existing literature by examining the relation between student characteristics and teacher language within elementary classrooms serving students with autism, documenting differences between general and special education classrooms.

### Teacher Language Within Classrooms Serving Students with Autism

To begin, we found that teachers in special education classrooms used relatively more language overall than teachers in general education classrooms, with much of their talk (69%) consisting of directives and close-ended questions that were directed at the individual student. Because we analyzed the language environment that each student experienced, we captured, at times, both teachers and paraprofessionals directing language at a student simultaneously within special education classrooms. This high frequency of competing verbal bids directed at individual students may be potentially concerning given the core communication challenges that differentiate students with autism (APA, 2000). That is, too much language or different features of talk directed simultaneously may overwhelm students with autism rather than afford them with rich language learning opportunities. Future research that includes measures of teacher language and student participation is needed, however, to better understand how teachers might best match their language to meet their students’ needs.

In addition, studies suggest that teachers’ use of directive language tends to be intrusive as it is often used to control, stop, or redirect student behavior (de Kruif et al., 2000; McWilliam et al., 2002; Williford et al, 2017). Increased directive language has been found to have detrimental effects on classroom active engagement for students with autism, jeopardizing the quality of the interaction and further intensifying the presence of challenging behaviors (Keen et al., 2005). Close-ended questions have also been found to hinder student participation and generativity within an exchange (e.g., Milburn et al., 2014). By framing questions in a close-ended manner, teachers may be providing more structure to interactions, yet they might also be limiting opportunities for their students think critically about the content and generate new, creative ideas and responses (Milburn et al., 2014)—which could potentially impact their developmental and educational outcomes (Connor et al., 2020).

Although we documented more language overall in special education classrooms, teachers in general education classrooms used a greater proportion of directives and close-ended questions with their students than all other language categories, consisting of over 60% of their talk. Teachers in both settings used relatively fewer language models and fill-ins—features of talk that help to scaffold interactions (Battaglia & Mcdonald, 2016). They also rarely asked students open-ended questions. Yet, open-ended questions have consistently been associated with active engagement and academic growth (Connor et al., 2020; Milburn et al., 2014) as well as increased student initiations and generative talk (Duke et al, 2011; Walsh, 2002) in students with and without autism. Taken together, these data suggest that the language environment that students with autism experience in classrooms, may be less than optimal—potentially limiting their opportunities to engage in rich exchanges that support learning and development.

### Conceptualizing and Measuring Teacher Language in Classrooms

We tested three models of teacher language drawn from a large, comprehensive literature base in order to understand the dimensionality of talk teachers directed toward their students with autism in general and special education classrooms. Although previous studies have conceptualized teacher language by pragmatic function (e.g., Walsh & Rose, 2013) or by teachers’ use of open-versus close-ended language (e.g., Walsh, 2002), we found that the construct was best represented by a unidimensional factor consisting of six features of talk (open-ended questions, language models, close-ended questions, directives, indirect requests, fill-ins). Our observation of a single underlying teacher language factor may be unique to students with autism, considering we observed high frequencies of directives and close-ended questions yet very low frequencies of other types of talk (i.e., open-ended questions; fill-ins). This pattern of talk could have impacted the overall dimensionality of the construct. In addition, we found that the unidimensional model of teacher language was consistent across general and special education classrooms, but the degree to which specific features of talk contributed to the latent factor varied between the settings. This provides evidence that the types of talk that teachers use with their students with autism manifest differently between general and special education classrooms. This finding also coincides with and helps to explain the differences we documented in the association between student characteristics and teacher language across the two settings.

### Student Characteristics and Teacher Language

Our data suggest that the characteristics of learners with autism are related to specific features of their teachers’ talk, yet these relations differ between general and special education classrooms. We documented that teachers’ use of directives within general and special education classrooms were related to autism symptom severity, suggesting that teachers used more directives with their students who exhibited more severe autism symptoms. In special education classrooms, teachers also used more directives with their students who exhibited greater cognitive and vocabulary impairment, and more open-ended questions with students who exhibited less severe autism symptoms. Whereas, teachers in general education classrooms asked more open-ended questions of their students who exhibited stronger expressive vocabulary skills. Finally, using SEM we found that teachers in general education classrooms used less language overall with their students who exhibited limited receptive vocabulary, yet this was not the case within special education classrooms. These findings are consistent with previous studies (e.g., Dykstra et al., 2013) conducted in preschool and clinical settings, providing evidence that students with autism experience differences in the amount and type of language they receive in classrooms, and this is, in part, due to their autism symptoms, expressive and receptive vocabulary, and cognitive skills. Although outside the scope of this study, there may be a number of reasons why teachers use different types of talk with their individual students. Nevertheless, these data might suggest a need for teachers to include scaffolds, modifications, materials, and/or other adaptations into classroom activities rather than rely on oral language, such as the use of directives and/or close-ended questions, for students with limited language and lower cognitive skills. However, this warrants further investigation.

### Strengths and Limitations

One of the primary contributions that this study makes to the existing literature is the sampling and analysis of classroom video observations at the student level, collecting one video observation for every participating student. By examining teacher language within general and special education classrooms, this study provides a snapshot of each student’s unique experience within his/her classroom. Hence, the large and heterogeneous sample of students with autism supports the overall generalizability of our findings to the larger population of students with autism in elementary classrooms. In addition, a broad range of reliable and valid standardized measures were used to characterize the sample, including “gold standard” diagnostic measures to confirm autism. Evaluation of teacher language was carried out using systematic observational methods, allowing us to capture nuances of talk during a continuous sampling of classroom activities. Inter-rater agreement between observers was calculated for each of the teacher language categories and indicated good agreement overall. Finally, the use of advanced methodological approaches, including a latent approach, further contributes to the richness and novelty of these data relative to the extant literature examining teacher language in classrooms serving students with autism.

This study has a few notable limitations. We examined teacher language within a systematically selected 15-min sample of classrooms activities. However, future research is needed to understand how representative or reflective the sampled time is of the entire 60-min video observation—an area of need within systematic observational measurement more broadly (Yoder et al., 2018). In addition, we used systematic observational methods and continuous sampling to examine each instance of teacher language, which provided a rich depiction of the classroom language environment. However, this detailed analysis was time-consuming and may have limited utility outside the research laboratory. Efforts focusing on using empirical evidence from studies such as this one to develop practical tools that gauge teacher language within classrooms could greatly advance the field. Although this study included a large sample of students with autism in general and special education classrooms, the sample size is considered fairly small for the analytical methods that were used. Therefore, we were unable to account for the nesting nature of the data when running the multi-group CFA. Future studies, which include students with and without autism, are needed to fully grasp how these findings can be applied to educational settings for elementary students with autism more broadly.

Furthermore, differences between classroom types regarding the proportion of students who are racially/ethnically diverse may have contributed some bias to the results and should be fully explored in future studies to better understand the intersectionality between classroom placement, student characteristics, and racial/ethnic background. Finally, we did not collect information on teacher/student ratio within general and special education classrooms or similarities and differences between teachers’ instructional approaches or teaching philosophies across educational settings. Hence, future studies are needed to understand the impact of adult support and varying evidence-based interventions (i.e., such as positive behavioral supports, ABA techniques, and cooperative learning opportunities) on the language environment.

### Implications for Research

By examining the factor structure of teacher language, this study provides initial empirical support for quantifying and measuring the amount and type of talk that teachers use with their students with autism in general and special education classrooms. Hence, this comprehensive examination of the classroom language environment provides a foundation for understanding specific features of teachers’ talk that may relate to (or hinder) student development and learning—an area of research that could help inform best practices for learners with autism. Findings from this study may also help to improve the consistency and accuracy of evaluating teacher language within classrooms. However, future research linking teacher language to student outcomes as well as examining the utility of a unidimensional model of teacher language across populations is needed.

In addition, the differences in teacher language that we documented between general and special education classrooms may have important implications for learners with autism. One potential concern is that the language environment within special education classrooms may not adequately prepare students for the linguistic and social pragmatic directives within general education classrooms (e.g., amount and types of talk). That is, inconsistent talk between general and special education classrooms may create an instructional barrier for learners with autism who transition between settings, as consistency between environments may provide the predictability needed to support generalization. While this is only speculation, our findings suggest that the language environment is important to consider when evaluating teachers and students within educational settings.

Finally, the links that we observed between teacher language and student characteristics (i.e., more directives used with students who exhibit more severe autism symptoms) suggest that students with more severe autism symptoms and those with co-occurring cognitive and/or vocabulary impairment may have limited access to a rich language environment that supports their learning and development. Hence, these findings raise a potential concern related to equity and highlight the need for continued research that focuses on understanding and evaluating educational practices for learners with autism. Efforts related to identifying and evaluating salient features of teachers’ talk that support student engagement and learning will offer insight into effective school-based practices while ensuring that all students with autism, including those with more severe impairment, are presented with equitable learning opportunities across educational settings.

## Appendix

Teacher language categories: definitions, examples, and coding specifications.

**Table:**

	Examples	Coding specifications
Open-ended questions		
Questions in which the answer is not predetermined by the teacher (Milburn et al., 2014). Open-ended questions do not have fixed answers so they elicit an infinite numbers of responses. Open-ended questions include “how” and “why” questions as well as other “wh” questions stated in a manner that encourages thinking and challenges students to generate their own thoughts and ideas (Connor et al., 2020; Sparapani et al., 2020)	*“What kind of games do you like to play?”* *“Why do you think the girl was so happy?”* *“Why do you think she would do that?”* *“Why did the town respond in such an angry way?”*	The number of instances and percentage of time that teachers ask students open-ended questions. Each code is marked to indicate whether the teacher directed the open-ended question toward the individual student or the group of students
Language models		
Contextual statements about what students are seeing or experiencing. Teachers model words and statements or expands on students’ contributions without requesting the student to respond. Teachers might make comments about objects or events within an activity/environment or talk about his/her experiences	*“I see a red marker.”* *“It looks very cold outside.”* *“Dinosaurs are huge. Scary!”* *“This is my favorite part of the book!”*	The number of instances and percentage of time that teachers model language or make contextual statements related to what the student (s) are seeing or experiencing. Each code is marked to indicate whether the teacher directed talk toward the individual student or the group of students
Close-ended questions		
Questions that are structured in a manner to elicit a specific response, often a single word or short fixed responses (Milburn et al., 2014). Close-ended questions consist of teachers’ use of choice questions, yes/no questions, and simple “wh” questions (Connor et al., 2020; Sparapani et al., 2020)	*“What is on the man’s head?”* *“Is that a cat?”* *“Do you want red or blue?”* *“Did you like that story?”*	The number of instances and percentage of time that teachers ask students close-ended questions. Each code is marked to indicate whether the teacher directed the close-ended question toward the individual student or the group of students
Directives		
Language used to direct or redirect student behavior to comply in a specific manner (de Kruif et al., 2000). Teachers’ use of language to demand the student to perform a given action or redirect the student’s behavior	*“Sit down.”* *“Get your book.”* *“Hands quiet.”* *“Push push the door open.”*	The number of instances and percentage of time that teachers direct students to comply in some manner. Each code is marked to indicate whether the teacher directed talk toward the individual student or the group of students
Indirect requests		
Directives stated in and indirect manner (often as a question) but are used to elicit a particular response. The teacher indirectly asks students to comply in a specific manner	*“Can you sit down?”* *“Did you get your book out?”* *“Will you come over here?”* *“Where are your hands supposed to be?”*	The number of instances and percentage of time that teachers indirectly ask students to comply in some manner. Each code is marked to indicate whether the teacher directed talk toward the individual student or the group of students
Fill-ins		
Sudden pauses used with practiced phrases and routines to encourage students to communicate the correct response. This also includes teachers’ use of exaggerated language to indicate that the student should take a conversational turn	*“100, 200, 300, 400, ____.”* *“Ready, set, _____.”* *“I want the _____.”* *“Time to check myyyy _____”*	The number of instances and percentage of time that teachers use fill-ins. Each code is marked to indicate whether the teacher directed talk toward the individual student or the group of students
